# Stomatal CO_2_ responsiveness and photosynthetic capacity of tropical woody species in relation to taxonomy and functional traits

**DOI:** 10.1007/s00442-017-3829-0

**Published:** 2017-03-04

**Authors:** Thomas B. Hasper, Mirindi E. Dusenge, Friederike Breuer, Félicien K. Uwizeye, Göran Wallin, Johan Uddling

**Affiliations:** 1 Department of Biological and Environmental Sciences, University of Gothenburg, PO Box 461, 405 30 Gothenburg, Sweden; 2 Department of Biology, University of Rwanda, University Avenue, PO Box 56, Huye, Rwanda

**Keywords:** Carbon dioxide, Transpiration, Leaf traits, Stomatal patterning, Tropical trees

## Abstract

Stomatal CO_2_ responsiveness and photosynthetic capacity vary greatly among plant species, but the factors controlling these physiological leaf traits are often poorly understood. To explore if these traits are linked to taxonomic group identity and/or to other plant functional traits, we investigated the short-term stomatal CO_2_ responses and the maximum rates of photosynthetic carboxylation (*V*_cmax_) and electron transport (*J*_max_) in an evolutionary broad range of tropical woody plant species. The study included 21 species representing four major seed plant taxa: gymnosperms, monocots, rosids and asterids. We found that stomatal closure responses to increased CO_2_ were stronger in angiosperms than in gymnosperms, and in monocots compared to dicots. Stomatal CO_2_ responsiveness was not significantly related to any of the other functional traits investigated, while a parameter describing the relationship between photosynthesis and stomatal conductance in combined leaf gas exchange models (*g*_1_) was related to leaf area-specific plant hydraulic conductance. For photosynthesis, we found that the interspecific variation in *V*_cmax_ and *J*_max_ was related to within leaf nitrogen (N) allocation rather than to area-based total leaf N content. Within-leaf N allocation and water use were strongly co-ordinated (*r* ^2^ = 0.67), such that species with high fractional N investments into compounds maximizing photosynthetic capacity also had high stomatal conductance. We conclude that while stomatal CO_2_ responsiveness of tropical woody species seems poorly related to other plant functional traits, photosynthetic capacity is linked to fractional within-leaf N allocation rather than total leaf N content and is closely co-ordinated with leaf water use.

**Electronic supplementary material:**

The online version of this article (doi:10.1007/s00442-017-3829-0) contains supplementary material, which is available to authorized users.

## Introduction

**Fig. 1:**
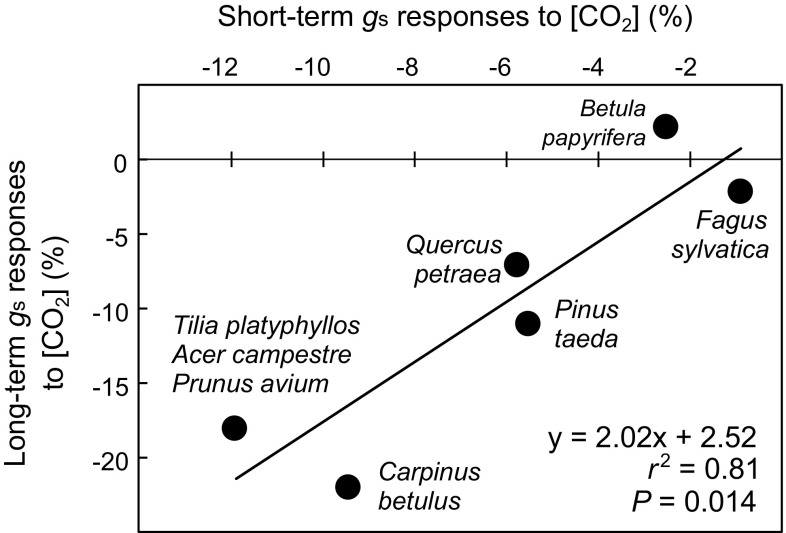
Relationship between the short-term response of *g*_s_ to elevated [CO_2_] [measured during short (hours) interruptions in CO_2_ enrichment] and the long-term effect of growth under elevated [CO_2_] on *g*_s_ in temperate forest free-air CO_2_ enrichment (FACE) experiments. Regression statistics are shown in the figure. Based on data from Cech et al. (2003), Keel et al. (2007), Maier et al. (2008), Domec et al. (2009), Onandia et al. (2011) and Tor-ngern et al. (2015)

Anthropogenic fossil fuel burning and land use change have increased the atmospheric carbon dioxide concentration [CO_2_] by over 40% (Ciais et al. 2013), with today’s concentration of over 400 μmol mol^−1^ being the highest in approximately 40 millions years (Frank et al. 2015). This large and rapid ongoing increase in [CO_2_] has profound impacts on land plants, which typically respond to altered [CO_2_] by increasing leaf photosynthesis (*A*) and decreasing stomatal conductance (*g*_s_; Ainsworth and Rogers 2007). However, the direct stomatal responses to a short-term increase in [CO_2_] vary greatly among species and experiments, from no change to 75% reduction in *g*_s_ at doubled compared to ambient [CO_2_] (Morison 1998). Understanding this variation is important, since the short-term stomatal CO_2_ response appears to be an important determinant for the long-term effect of growth in elevated [CO_2_] on *g*_s_ under field conditions. In so called free-air CO_2_ enrichment (FACE) experiments with trees, the interspecific variation in long-term (years) effects of growth under elevated [CO_2_] on *g*_s_ was significantly and positively related to the variation in short-term stomatal CO_2_ responsiveness, measured as the effect of short (hours) interruptions in CO_2_ enrichment on sap flow (Cech et al. 2003; Keel et al. 2007; Tor-ngern et al. 2015) or leaf *g*_s_ (Maier et al. 2008; Domec et al. 2009; Onandia et al. 2011) in some of these experiments (Fig. 1; *r* ^2^ = 0.81; *P* = 0.014). Tree species that exhibit a pronounced direct stomatal closure response to a short-term increase in [CO_2_] are thus also likely to develop a long-term *g*_s_ reduction in the field. Therefore, a better understanding of the factors that control the large natural variation in short-term stomatal CO_2_ responses would provide indication of which plant species and groups that are likely to experience large increases in water-use efficiency in future higher [CO_2_] scenarios.

Both the short-term stomatal CO_2_ response and the long-term effect of growth under elevated [CO_2_] on *g*_s_ have been found to be weaker in gymnosperms compared to angiosperms (Medlyn et al. 2001; Brodribb et al. 2009). Brodribb et al. (2009) even suggested that gymnosperms lack a primary stomatal CO_2_ response, while others have found that this is not the case (Haworth et al. 2013). To our knowledge, there has been no study designed to explore possible taxonomic patterns in the large interspecific variation in stomatal CO_2_ responsiveness among different angiosperm taxa. Furthermore, possible functional determinants of the interspecific variation in stomatal CO_2_ responsiveness have also been poorly explored. If stomatal CO_2_ responses are linked to physiological, chemical or structural plant traits for which more knowledge is available, this could facilitate future trait-based modelling of plant water-use under rising [CO_2_] (Van Bodegom et al. 2012). For example, it is possible that (but unknown if) stomatal CO_2_ responsiveness is linked to stomatal density since, in general, plants with high stomatal density have more efficient stomatal regulation (Franks et al. 2009). It is also plausible that stomatal CO_2_ responsiveness is linked to hydraulic traits since plants with high hydraulic conductance often exhibit tight stomatal control over transpiration to avoid severe cavitation (Bond and Kavanagh 1999). Since stomatal regulation is an energy consuming process, CO_2_ sensitivity may also be linked to indicators of leaf metabolic activity, such as photosynthetic capacity.

Most land-surface and ecosystem models apply combined stomatal–photosynthesis models (Ball et al. 1987; Leuning 1995; Medlyn et al. 2011), in which plant carbon and water vapor fluxes are linked as they both pass through plant stomata. The empirical slope parameter of the combined stomatal–photosynthesis model (*g*_1_), which is inversely proportional to leaf water use efficiency (at constant environmental conditions; Medlyn et al. 2011), has been determined for a large number of plant functional types and in different biomes (Lin et al. 2015). In angiosperm trees, it was found that *g*_1_ was positively related to wood density, reflecting a higher cost of wood construction per unit water transported in species with higher wood density (Lin et al. 2015). However, the relationship with wood density was quite weak (*r* ^2^ = 0.21; Lin et al. 2015) and when gymnosperms were included it was not significant at all. There is thus a need to further explore the relationship of *g*_1_ with wood density and other plant traits, such as hydraulic conductance. Hydraulic conductance and *g*_s_ are often co-ordinated in trees (Brodribb and Jordan 2008) and since high hydraulic conductance essentially reduces the carbon cost of water use (Prentice et al. 2014) it is plausible that (but unexplored if) *g*_1_ is positively related to hydraulic conductance. With respect to taxonomic patterns, there are typically distinct differences in water-use strategy between angiosperm and gymnosperm trees, with angiosperms typically exhibiting higher *g*_s_, hydraulic conductance and *g*_1_ at the expense of considerably smaller hydraulic safety margin compared to gymnosperms (Choat et al. 2012; Lin et al. 2015).

There is also considerable uncertainty with respect to the variation in the maximum rates of photosynthetic carboxylation (*V*_cmax_) and electron transport (*J*_max_) among plant species in general (Ali et al. 2015) and tropical tree species in particular (Dusenge et al. 2015). Current vegetation models typically base their values of *V*_cmax_ and *J*_max_ on area-based total leaf N content (e.g., Zaehle et al. 2005; Rogers 2014). However, a recent global meta-analysis found that *V*_cmax_ and *J*_max_ were more closely related to within-leaf nitrogen (N) allocation than to total leaf N content (Ali et al. 2015), as also observed in two studies with tropical tree species (Coste et al. 2005; Dusenge et al. 2015). Part of the reason for why interspecific variation in photosynthetic capacity is often poorly related to total leaf nutrient content may be linked to differences in within-leaf N allocation among species with different successional strategies. It is well established that early-successional species invest more leaf N into N-rich molecules involved in photosynthesis and respiration compared to late-successional tree species, which typically have larger fractional N investments in rather N-poor structural compounds (Raaimakers et al. 1995; Valladares and Niinemets 2008). Dusenge et al. (2015) further suggested that there is a trade-off in how the photosynthetic leaf N is invested, with pioneer species making higher relative investments into compounds maximizing photosynthetic carboxylation and bioenergetics (i.e., *V*_cmax_ and *J*_max_) and climax species investing more into compounds involved in light-harvesting. However, that study included a rather small number of species and this hypothesis, therefore, needs to be further evaluated by field data.

Tropical forests have a fundamental role in the global carbon and water cycles and in controlling the rate of climate change (Lewis 2006). They account for about a third of the global terrestrial primary production (Beer et al. 2010) and their evapotranspiration regulates regional temperature and precipitation and runoff patterns (Bonan 2008). Given all the challenges associated with research in tropical forests (e.g. large stature, biological complexity and logistics), much less is known about stomatal regulation and photosynthesis of trees in these ecosystems compared to temperate forests (Kattge et al. 2009). To improve the poor understanding of the phylogenetic and functional controls of the large interspecific variation in stomatal CO_2_ responsiveness and photosynthetic capacity among tropical woody species, we examined physiological, chemical and structural plant traits in an evolutionary broad cross-section of tropical woody seed plant species in a common garden experiment in Rwanda. The specific objectives were to answer the following three research questions: (1) Does the stomatal response to a short-term increase in [CO_2_] vary among major taxonomic groups? (2) What functional characteristics can explain the interspecific variation in stomatal behavior (i.e. in the short-term CO_2_ response and *g*_1_)? (3) Is the interspecific variation in photosynthetic capacity controlled by differences in within-leaf nutrient allocation rather than by differences in total area-based leaf nutrient content?

## Materials and methods

### Study sites and plant material

From February 27 until March 25 in 2014, data were collected on mature (having reached reproductive stage) woody plants of 21 species growing at four locations in the surroundings of Butare, Huye district, Rwanda, central Africa: Rwanda Agriculture Board (RAB) Ruhande Arboretum (“Arboretum” hereafter; 2°36′55.2′′S, 29°44′53.8′′E; 1700 m asl; 17 species), Ruhande Fisheries Research Station (2°36′22.1′′S, 29°45′29.7′′E; 1650 m asl; one species), Marist Missionary Sisters of the Society of Mary’s garden (2°36′32.1′′S, 29°44′37.6′′E; 1700 m a.s.l.; two species) and RAB Rubona station (2°38′57.1′′S, 29°45′54.2′′E; 1690 m a.s.l.; one species). The Arboretum was established in 1934 and since then has gathered 227 tree species (50 native to Rwanda) planted, in most part, as replicated (*n* = 3) monospecific 50 × 50 m plots within its 200 ha plantation area. Out of the 21 species investigated five were native to Africa and 16 were exotic (Table 1).

**Table 1:** Tree species description and their plot location at the Ruhande Arboretum

Group		Order	Family	Species	Origin	Plot	Mean DBH (cm)	Leaf longevity	Leaf shape	Stand planted (year)
Gymnosperms		Pinales	Araucariaceae	*Araucaria angustifolia* (Bertol.) Kuntze	Exotic	47	19.2	EG	NL	1938
		Pinales	Cupressacaea	*Cupressus lusitanica* Mill.	Exotic	144	36.6	EG	NL	1948
		Pinales	Pinaceae	*Pinus patula* Schlecht. & Cham.	Exotic	48	66.9	EG	NL	1959
		Pinales	Podocarpaceae	*Podocarpus latifolius* (Thunb.) R.Br. ex Mirb.	Native	43	19.7	EG	NL	1948
		Pinales	Podocarpaceae	*Podocarpus falcatus* (Thunb.) R.Br. ex Mirb	Native	2	39.2	EG	NL	1955
Angiosperms										
Monocots (Commelinids)		Arecales	Arecaceae	*Phoenix reclinata* Jacq.	Native	a	22.0	EG	BL	~2004
		Poales	Poaceae	*Dendrocalamus giganteus* Munro	Exotic	293	6.4	EG	BL	2005
		Poales	Poaceae	*Bambusa vulgaris* Schrad.	Exotic	26	5.0	EG	BL	1934
		Zingiberales	Heliconiaceae	*Heliconia rostrata* Ruiz & Pavon	Exotic	b	2.2	EG	BL	~2009
		Zingiberales	Musacea	*Musa sapientum* L.	Exotic	b	5.6	EG	BL	~2009
Dicots										
Rosids		Malphigiales	Chrysobalanaceae	*Macaranga kilirnandscharica* (Pax.)	Native	224	8.0	EG	BL	2008
		Rosales	Rosaceae	*Prunus caretta*	Exotic	65	15.0	EG	BL	1943
		Myrtales	Myrtaceae	*Eucalyptus maculata* (Hook.) K.D. Hill & L.A.S. Johnson	Exotic	6	15.7	EG	BL	1934
		Sapindales	Meliaceae	*Carapa grandiflora* Sprage	Native	11	19.9	EG	BL	1948
		Sapindales	Meliaceae	*Cedrela serrata* Royle	Exotic	111–112	22.2	EG	BL	1945
		Malvales	Malvaceae	*Brachychiton acerifolius* (A.Cunn. Ex G.Don) Macarthur & C. Morre	Exotic	75	14.9	DE	BL	1943
Asterids		Lamiales	Bignoniaceae	*Jacaranda rnirnosifolia* D.Don	Exotic	8	42.4	DE	BL	1934
		Lamiales	Boraginaceae	*Cordia alliodora* (Ruiz & Pay.) Oken	Exotic	30	9.1	EG	BL	1984
		Lamiales	Oleaceae	*Ligustrum lucidum* W. T.Aiton	Exotic	67	5.1	EG	BL	1943
		Solanales	Solanaceae	*Cyphornandra betaceae* Cay.	Exotic	c	2.7	EG	BL	2012
		Asterales	Asteraceae	*Tithonia diversifolia* (Hemsl.) A.Gray	Exotic	37	1.8	EG	BL	~2008

*DBH* diameter at breast high, *EG* evergreen, *DE* deciduous, *NL* needle leaf, *BL* broadleaf)

a Fisheries Research Station Adjacent to the Arboretum

b Marist Missionary Sisters of the Society of Mary’s Garden

c RAB Rubona station

The nearest meteorological station is located ca. 2 km from the Arboretum, showing for the period 2006–2013 mean day and night air temperatures of 20.8 and 17.1 °C, respectively, average humidity of 74%, and mean annual rainfall of 1231 mm. The climate of the region is characterized as tropical humid, with 1.5 °C difference between the warmest and coolest months. Precipitation exhibits some seasonal variation, with the highest and lowest amounts occurring in March–May and June–August, respectively. Additional meteorological information can be found in Nsabimana et al. (2009) and Vårhammar et al. (2015).

The 21 species were selected to represent a broad evolutionary cross-section of seed plant species, representing four major lineages of seed plants: gymnosperms, monocots [commelinids], rosids and asterids (Table 1). Of the selected species, 16 were silviculturally or agriculturally important exotics, while five were native tropical species. All species exhibited a woody stem and plants investigated were older than two years. In this study, we define wood as tissue produced of secondary xylem, including highly lignified xylem tissue in secondary vascular bundles in monocots. The focus on tropical woody species minimized sources of variation related to growth form and climate of origin, thereby increasing the chance of finding possible taxonomic patterns. For each species, measurements were done on five individual plants, measuring leaf gas exchange on one fully developed leaf of each individual (except for *Phoenix reclinata*, where two and three leaves were measured in each of two different plants).

### Gas exchange measurements

Leaf gas exchange measurements were conducted in situ between 09:00 and 15:00 h using two portable photosynthesis systems (LI-6400 and LI-6400XT with the 2 × 3 cm standard leaf chamber and LI-6400-02B LED light source; Li-Cor Inc., Lincon, NE, USA). For each species, five fully developed, visually healthy, sun-exposed leaves (one per plant) were selected and measured for the response of net photosynthetic rate (*A*_n_) to eight different [CO_2_] [so called *A*-*C*_i_ curves; *C*_i_ = leaf intercellular (CO_2_)]. In large trees, the measured leaves were attached to lower sun-exposed branches by the edge of the stand. All measurements were conducted at a photosynthetic photon flux density (PPFD) of 1800 µmol m^−2^ s^−1^ and a leaf temperature of 25 °C. Following the *A*-*C*_i_ curve measurements, direct responses of *g*_s_ to a short-term change in leaf chamber [CO_2_] (600 vs. 400 μmol mol^−1^) were measured on the same leaves, at the same leaf temperature and PPFD. Measurements were taken every minute and the steady-state *g*_s_ at each [CO_2_] was recorded after *g*_s_ values had stabilized for at least 15 min and changed ≤2% within a 5 min period. The order of the [CO_2_] applied was randomized for the first leaf of a species, and then alternated for the remaining leaves of that species. For each leaf, the leaf-to-air vapour pressure deficit (*D*) was kept constant (± 0.03 kPa) around a leaf-specific value in the range 1.15–1.80 kPa during the entire measurement.

Shoots of needle-leaf species were also measured using the standard leaf chamber. Needles were removed at the points where the shoot axis passed the chamber gasket. Needles were scanned (Epson V200 Perfection, Epson America Inc., USA) and their projected surface area were estimated using WinRHIZO analysis software (WinRHIZO, Regent Instruments Inc., Canada), and leaf and needle temperatures were calculated using energy balance equations.

### Photosynthesis model parameterization

The photosynthesis model by Farquhar et al. (1980), with modifications of photosynthetic temperature dependencies by Bernacchi et al. (2001), was used to parameterize *V*_cmax_ and *J*_max_ from *A*-*C*_i_ curve data by the least squares method. The rates of *V*_cmax_-limited net photosynthesis (*A*_c_) and *J*_max_-limited net photosynthesis (*A*_j_) were calculated using:

1 $$ A_{c} = \frac{{V_{{{\text{c max}}  }}  (C_{\text{i}} - \varGamma^{*} )}}{{C_{\text{i}} + K_{\text{c}} \left( {1 + \frac{O}{{K_{\text{o}} }}} \right)}} - R_{\text{d}} $$

and

2 $$ A_{\text{j}} = J\frac{{C_{\text{i}} - \varGamma^{*} }}{{4 C_{\text{i}} + 8\varGamma^{*} }} - R_{\text{d}} $$

where *K*_c_ and *K*_o_ are Michaelis–Menten constants for CO_2_ and O_2_, respectively; Γ* is the [CO_2_] at which the carboxylation reaction of Rubisco equals the oxygenation reaction; *R*_d_ is the non-photorespiratory CO_2_ release in the light; and *J* is the rate of electron transport. For *K*_c_, *K*_o_ and Γ*, the values at 25 °C as well as the temperature sensitivities were taken from Bernacchi et al. (2001). The internal leaf conductance for CO_2_ was not estimated and, therefore, ‘apparent’ *V*_cmax_ and *J*_max_ values are reported, based on *C*_i_ rather than on the [CO_2_] at the chloroplast. The uncertainty of the values of the curvature of the light-response (0.9) and quantum yield of electron transport (0.3 mol electrons mol^−1^ photons) used when calculating *J*_max_ from *J* has only a minor effect on the estimated value of *J*_max_ (Medlyn et al. 2002). Values of *V*_cmax_, *J*_max_ and *R*_d_ were determined simultaneously with the only a priori restriction made to the *A*−*C*_i_ fitting that data points with *C*_i_ below 100 µmol mol^−1^ were forced to be *V*_cmax_-limited. The discontinuity of the transition from *V*_cmax_- to *J*_max_-limitation in the *A*−*C*_i_ curve was not smoothed out. *J*_max_ values were reported only if the *A*_j_ limited part of the *A*−*C*_i_ curve had at least two data points, or from one single data point if *A*_j_ was at least 10% lower than *A*_c_ at the *C*_i_ value of that data point. The latter was the case in nine leaves only and the *J*_max_ value of these leaves were similar to those of the other leaves. Light-saturated net photosynthesis at a *C*_i_ of 280 µmol mol^−1^ (*A*_n280_; i.e. assuming a *C*_i_ that is 70% of an ambient CO_2_ concentration of 400 µmol mol^−1^) was calculated based on the fitted photosynthesis model for each leaf.

Leaf-specific values of *g*_1_ were calculated from the steady-state leaf gas exchange measurement at 400 μmol mol^−1^ CO_2_ concentration of the air, according the model for optimal stomatal conductance given in Medlyn et al. (2011):

3 $$ g_{\text{s}} \approx g_{0} + 1.6\left( {1 + \frac{{g_{1} }}{\sqrt D }} \right)\frac{{A_{\text{{n} }}}}{{C_{\text{a}} }} $$

where *g*_0_ is the *g*_s_ at zero *A*_n_, 1.6 is the ratio of the diffusivities of water vapour and CO_2_, and *C*_a_ is the [CO_2_] of the air surrounding the leaf (µmol mol^−1^). Values of *g*_0_ are often small (Medlyn et al. 2011) and were assumed to be zero in the present study. The model parameter *g*_1_, which is the key parameter of the optimal stomatal conductance model and inversely related to water use efficiency, could thus be analytically calculated for each individual leaf.

### Additional leaf traits

After the gas exchange measurements, epidermal impressions of around 2 cm^2^ of the center of the left part of the abaxial leaf surface were made from all measured leaves except *Tithonia diversifolia* (due to its hairy leaf surface) to determine stomatal density and length. A thin layer of nail varnish was carefully peeled off after 10 min drying, and was gently placed over a microscope slide and covered and sealed by a cover slip. Six photos of evenly distributed areas of each peel where taken using a microscope (Zeiss, Axio Scope.A1, Germany) equipped with a digital camera (Zeiss, AxioCam MRm, Germany) using 100 × magnification. The photos were treated for higher contrast and definition using ImageJ 1.48v software before measurements of stomatal density and guard cell length. Stomatal density was estimated by calculating the average number of stomata per mm^2^ from the six photos taken from each peeling, except in *Macaranga kilimandcharica* where photos could be used from only one of the five peels. Stomatal length was estimated by calculating the average of 30 randomly chosen guard cell pairs from three of the six photos taken (10 from each photo). The estimates of stomatal density and length were used to calculate the maximal *g*_s_ that was anatomically possible (*g*_smax_) according to Franks and Beerling (2009):

4 $$ g_{\text{smax}} = \frac{{d_{\text{w}} / v* \rho * a_{\hbox{max} } }}{{l + \pi /2 \sqrt {a_{\hbox{max} } /\pi } }} $$

where *d*_w_ is the diffusivity of water vapour (0.26 m^2^ s^−1^), *v* is the molar volume of air (24.47 dm^3^ mol^−1^), *ρ* is the number of stomata per m^2^, *a*_max_ is the area of a fully open stomatal pore (m^2^) and *l* is the depth of stomatal pore (m). Assumptions used in the calculation of stomatal pore length, width and depth in relation to guard cell length were the same as in Franks and Beerling (2009).

Leaf length, width and thickness were recorded. Leaf discs of known area were punched from each leaf, avoiding major veins, to determine leaf mass per unit area (LMA) and leaf N and phosphorus (P) content. Leaves of species with needles (*Cupressus lusitanica*, *Pinus patula, Araucaria angustifolia*, *Podocarpus latifolius*, *Podocarpus falcatus*) or small leaves (*Jacaranda mimosifolia*) were scanned (Epson Expression 1600, Epson America Inc., CA, USA) and projected leaf surface area was determined using WinRhizo software (2007, Regents Instruments, Canada). The collected leaf material was dried to constant weight at 70 °C before dry mass was recorded. The leaf discs and needles were milled using a ball mill (Retsch MM301, Haan, Germany) and leaf N concentration was determined using an elemental analyser (Europe EA-GSL, Sercon Ltd., Crewe, UK) coupled to a stable isotope ratio mass spectrometer (20-22, Sercon Ltd. Crewe, UK). Leaf P content was determined for three leaves per species by first treating samples with the Kjeldahl method and later analyzing them with a continuous flow automated analyzer (SEAL Analytical, AutoAnalyzer 3HR, Norderstedt, Germany). Leaf chlorophyll content was determined by extraction of leaf samples in 80% acetone and subsequent filtering and spectrophotometric absorbance measurement, as described by Uddling et al. (2007).

Immediately after the gas exchange measurements, leaves were collected and leaf water potentials (*Ψ*_leaf_, MPa) were measured using a Scholander type pressure chamber (SAPS II Model 3115, Soilmoisture Equipment Corp., Santa Barbara, CA, USA). In addition, pre-dawn *Ψ*_leaf_ measurements were done in the last day of the measurement campaign (except for *Phoenix reclinata*, *Musa sapientum*, *Heliconia rostrata* and *Cyphomandra betaceae*). The measured values of pre-dawn *Ψ*_leaf_ were high, and this variable likely did not vary much over the 1 month measurement campaign since precipitation was quite high and temperature similar in both March (109 mm/19.5 °C; measurement month) and February 2014 (89 mm/19.2 °C; preceding month). The evening before the pre-dawn *Ψ*_leaf_ measurements, one leaf per tree was kept in darkness inside a water vapour saturated Ziploc plastic bag covered in aluminium foil. At pre-dawn on the following day (ca. 05:00 h) the plastic bags were collected and pre-dawn *Ψ*_leaf_ was measured and used as an estimate of soil water potential (*Ψ*_soil_). From data of *Ψ*_soil_ (i.e., pre-dawn *Ψ*_leaf_), leaf transpiration (*E*), leaf temperature and *Ψ*_leaf_, leaf area-specific plant hydraulic conductance (*K*_p_) was determined according to:

 5 $$ K_{\text{p}} = \frac{{\tilde{v}E}}{{\psi_{\text{soil}} - \psi_{\text{leaf}} }} $$

where *ṽ* is the relative temperature dependent kinematic viscosity of water (set to 1 at 20 °C; dimensionless). Values of *ṽ* were calculated using leaf-specific values of leaf temperature, which may be a fair simplification since the leaf accounts for about half of the whole-plant hydraulic resistance (Sack and Holbrook 2006). Soil water potential values for the three species lacking pre-dawn data were taken as the mean value across all other species.

A 3 cm piece of wood on a branch close to the measured leaf was cut and its volume (without the bark) was measured by immersion in a volumetric cylinder on an analytical balance (except for *H. rostrata* and *M. sapientum* which have pseudostem). All pieces of wood were later dried to constant weight at 70 °C and wood mass and density were determined.

The fractional leaf N investments were determined for the following components of the photosynthetic apparatus: Rubisco (N_R_); bioenergetics, including coupling factors, electron carriers except for photosystems, and Calvin-Benson cycle enzymes except for Rubisco (N_B_); and light-harvesting complexes and photosystems (N_LH_). The N_R_ was estimated using the equation and parameters provided by Niinemets and Tenhunen (1997):

6 $$ {\text{N}}_{\text{R}} = \frac{{0.16 V_{\text{cmax}} }}{{{\text{N}}_{\text{a}} V_{\text{cr}} }} $$

where *V*_cmax_ is the maximum rate of carboxylation, 0.16 converts Rubisco to N [g N in Rubisco (g Rubisco)^−1^] and *V*_cr_ the specific activity of Rubisco at 25 °C [20.78 μmol CO_2_ (g Rubisco)^−1^ s^−1^]. The N_B_ was estimated as:

7 $$ {\text{N}}_{\text{B}} = \frac{{J_{\text{max}} }}{{{\text{N}}_{\text{a}} 156 \times 8.06}} $$

where it is assumed that N in bioenergetics is proportional to *J*_max_, that 156 is the ratio of electron transport to cytochrome f content in mol mol^−1^ s^−1^ and that 8.06 is the amount of cytochrome f per unit N in bioenergetics in μmol g^−1^ (Evans and Seemann 1989; Niinemets and Tenhunen 1997). The sum of N_R_ and N_B_ (N_R+B_) was used as a measure of leaf N in compounds determining the maximum photosynthetic rate, i.e. photosynthetic capacity. The N_LH_ was estimated according to Evans and Poorter (2001) as:

8 $$ {\text{N}}_{\text{LH}} = \frac{{0.0155 \times 41 {\text{Chl}}}}{{{\text{N}}_{\text{a}} }} $$

where Chl is the area-based chlorophyll content (g m^−2^), 0.0155 is the mass fraction of one mole N to one mole chlorophyll, and 41 is the N content per unit chlorophyll in light-harvesting complexes and photosystems in sun-exposed leaves in mol mol^−1^.

### Statistical analysis

One-way analysis of variance (ANOVA) with Scheffe’s posthoc test was performed using SPSS 21 software (IBM, New York, USA) to test whether structural, chemical and physiological characters differed significantly among the studied taxonomic groups. No heterogeneity of variance was found according to Cochran’s C test, but in two cases where data distributions were skewed (stomatal density and LMA), log-transformation was performed prior to ANOVA tests. Linear regression and Pearson’s correlation statistics to test the significance of relationships between the measured structural, chemical and physiological variables were also performed using SPSS 21. In three cases there were duplicate species represented within the same family (Podocarpaceae, Poacea and Meliaceae; Table 1). Replacing species-specific data of these six species with the family-specific mean values did not change the significances of any of the group comparisons or regressions of the study (data not shown).

## Results

### Taxonomic group comparisons

**Fig. 2:**
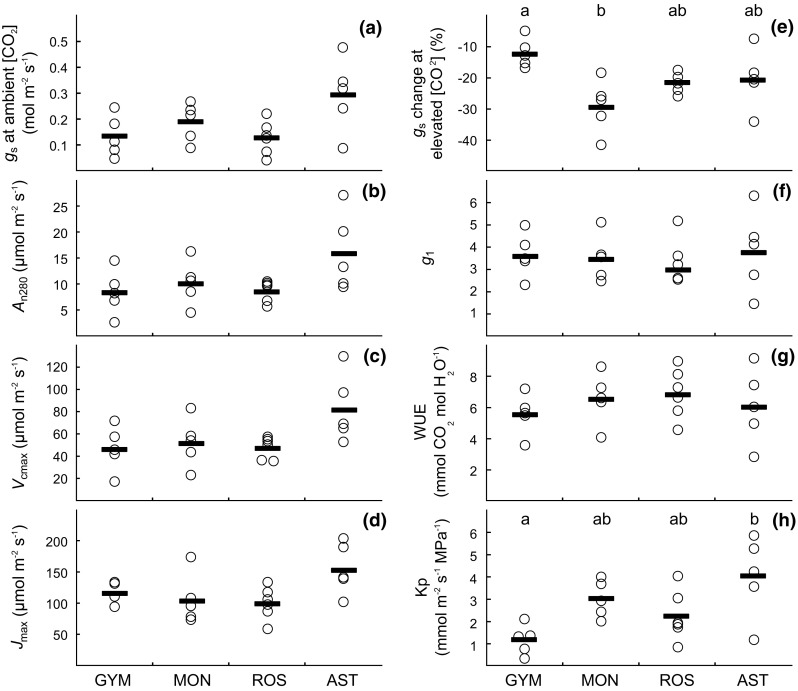
Physiological traits in different taxonomic groups: **a** stomatal conductance (*g*_s_) at ambient [CO_2_]; **b** net photosynthetic rate at 280 µmol mol^−1^ intercellular [CO_2_] (*A*_n280_); **c** maximum rate of carboxylation (*V*_cmax_); **d** maximum rate of electron transport (*J*_max_); **e** short-term *g*_s_ response to increased [CO_2_] (600 vs. 400 μmol mol^−1^); **f** *g*_1_ (see Eq. 3); **g** water use efficiency (WUE); and **h** leaf area-specific plant hydraulic conductance (*K*_p_). Each *data point* represents the mean value of a species and *thick black lines* represent mean values of the taxonomic groups. *GYM* gymnosperms; *MON* monocots; *ROS* rosids; and *AST* asterids

Values of *g*_s_ at 400 µmol mol^−1^ [CO_2_] did not significantly differ among taxonomic groups, with asterids having the highest mean but also the highest variation (Fig. 2a). Similarly, neither *A*_n280_ nor *V*_cmax_ or *J*_max_ differed significantly among these groups (Fig. 2b–d). All taxonomic groups exhibited significant stomatal closure responses to a short-term increase in [CO_2_], (Fig. 2e). Gymnosperms had a significantly weaker response (−12%) than monocots (−29%; *P* = 0.011) while asterids and rosids were intermediate and did not significantly differ from any other group. At a higher taxonomic level, stomatal CO_2_ responsiveness was stronger in angiosperms compared to gymnosperms and in monocots compared to dicots (Online Resource 1). The different taxonomic groups had similar values of *g*_1_ and WUE, again with asterids exhibiting the highest variation among species (Fig. 2f–g). Values of *K*_p_ varied greatly both among and within taxonomic groups, with gymnosperms having significantly lower *K*_p_ than asterids (*P* = 0.019; Fig. 2h).

**Fig. 3:**
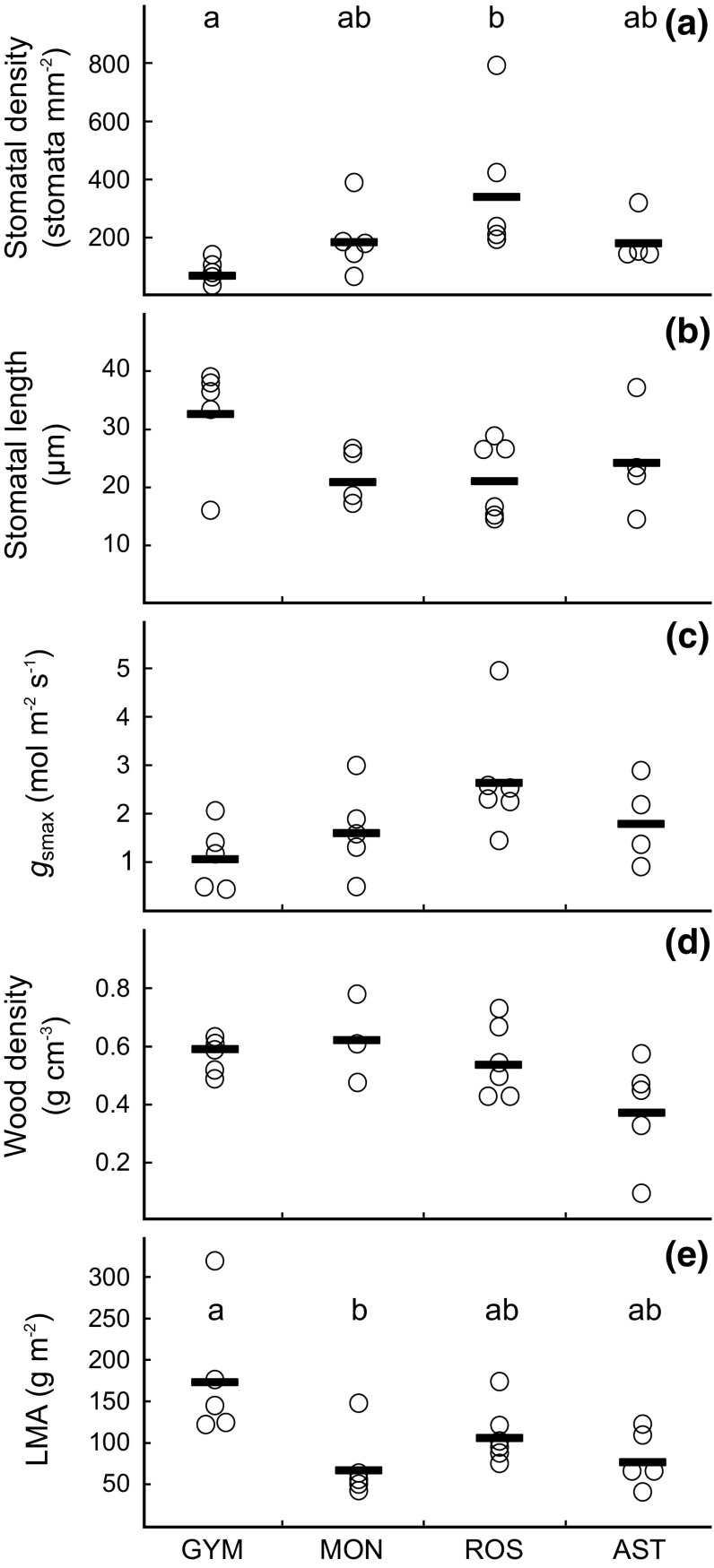
Structural traits in different taxonomic groups: **a** stomatal density; **b** stomatal length; **c** maximum stomatal conductance (*g*_smax_) calculated from stomatal density and length data (see Eq. 4); **d** wood density; and **e** leaf mass per unit area (LMA). Different letters represent significant difference between groups (*P* ≤ 0.05). Each *data point* represents the mean value of a species and *thick black lines* represent mean values of the taxonomic groups. *GYM* gymnosperms, *MON* monocots, *ROS* rosids, *AST* asterids

Stomatal density and stomatal length exhibited large variation both within and among taxonomic groups (Fig. 3a, b). Stomatal density was lower in gymnosperms than in rosids (*P* = 0.007), while stomatal length did not differ among taxonomic groups. Values of *g*_smax_ (determined from stomatal density and stomatal length data) and wood density varied greatly among species but did not significantly differ among groups (Fig. 3c–d). Gymnosperms had significantly higher LMA than monocots (*P* = 0.017) and marginally significantly higher LMA than asterids (*P* = 0.051; Fig. 3e).

Leaf area-based N and P contents (N_a_ and P_a_) did not significantly differ among taxonomic groups, but there were several differences in leaf mass-based concentrations (Table 2). Leaf mass-based N concentration (N_m_) was significantly lower in gymnosperms than in monocots (*P* = 0.043) and asterids (*P* = 0.004; Table 2), while leaf mass-based P (P_m_) did not significantly differ among taxonomic groups. Leaf mass-based C (C_m_) concentration was significantly lower in monocots than in gymnosperms (*P* = 0.003) and rosids (*P* = 0.023). The N:P ratio did not significantly differ among plant groups. Leaf area based chlorophyll content was significantly higher in gymnosperms than in monocots, rosids and asterids (*P* = 0.001, *P* = 0.014 and *P* = 0.007, respectively; Table 2).

**Table 2:** Chemical leaf trait values

Group	Family	Species	N_m_ (%)	P_m_ (%)	C_m_ (%)	N_a_ (g m^−2^)	P_a_ (g m^−2^)	N_m_:P_m_ (%)	Chlorophyll (g m^−2^)
Gymnosperms	Araucariaceae	*Araucaria angustifolia*	1.65 ± 0.15	0.15 ± 0.03	45.13 ± 0.78	1.97 ± 0.41	0.18 ± 0.04	10.67 ± 1.10	0.68 ± 0.06
	Cupressacaea	*Cupressus lusitanica*	1.26 ± 0.10	0.14 ± 0.03	45.99 ± 1.04	3.98 ± 0.49	0.45 ± 0.10	8.83 ± 1.32	0.85 ± 0.05
	Pinaceae	*Pinus patula*	1.59 ± 0.20	0.16 ± 0.00	46.45 ± 1.33	2.74 ± 0.36	0.27 ± 0.00	10.06 ± 0.33	0.77 ± 0.19
	Podocarpaceae	*Podocarpus latifolius*	1.62 ± 0.09	0.16 ± 0.01	45.59 ± 1.55	2.29 ± 0.57	0.23 ± 0.05	10.18 ± 1.15	0.59 ± 0.10
	Podocarpaceae	*Podocarpus falcatus*	1.79 ± 0.12	0.17 ± 0.02	43.42 ± 1.22	2.16 ± 0.18	0.21 ± 0.01	10.52 ± 0.98	0.57 ± 0.11
Mean			1.58 ± 0.17^A^	0.16 ± 0.01	45.31 ± 1.02^A^	2.63 ± 0.71	0.27 ± 0.09	10.05 ± 0.64	0.69 ± 0.10^A^
Monocots	Arecaceae	*Phoenix reclinata*	1.90 + 0.47	0.16 ± 0.01	42.84 ± 0.48	2.75 ± 0.73	0.23 ± 0.02	12.03 ± 0.66	0.32 ± 0.16
	Poaceae	*Dendrocalamus giganteus*	3.52 ± 0.12	0.15 ± 0.02	39.67 ± 2.46	1.39 ± 0.36	0.06 ± 0.03	23.93 ± 2.67	0.39 ± 0.07
	Poaceae	*Bambusa vulgaris*	2.99 ± 0.08	0.16 ± 0.00	40.05 ± 1.42	1.43 ± 0.24	0.08 ± 0.01	18.52 ± 0.58	0.37 ± 0.01
	Heliconiaceae	*Heliconia rostrata*	3.42 ± 0.41	0.29 ± 0.05	42.44 ± 1.27	1.81 ± 0.49	0.15 ± 0.04	11.92 ± 3.72	0.20 ± 0.05
	Musacea	*Musa sapientum*	3.05 ± 0.20	0.29 ± 0.05	40.33 ± 0.98	1.85 ± 0.24	0.17 ± 0.02	10.60 ± 2.79	0.18 ± 0.03
Mean			2.98 ± 0.56^B^	0.21 ± 0.06	41.07 ± 1.28^B^	1.85 ± 0.48	0.14 ± 0.06	15.40 ± 4.98	0.29 ± 0.09^B^
Rosidss	Chrysobalanaceae	*Macaranga kilirnandscharica*	2.07 ± 0.08	0.11 ± 0.02	43.17 ± 0.98	1.77 ± 0.27	0.10 ± 0.01	18.28 ± 2.91	0.40 ± 0.05
	Rosaceae	*Prunus caretta*	2.25 ± 0.38	0.29 ± 0.03	44.45 ± 1.31	2.22 ± 0.30	0.28 ± 0.04	7.86 ± 1.50	0.35 ± 0.06
	Myrtaceae	*Eucalyptus maculata*	1.28 ± 0.10	0.06 ± 0.01	45.21 ± 1.56	2.19 ± 0.28	0.11 ± 0.02	20.33 ± 2.75	0.44 ± 0.11
	Meliaceae	*Carapa grandiflora*	1.98 ± 0.29	0.11 ± 0.03	42.52 ± 1.59	2.34 ± 0.24	0.13 ± 0.04	17.63 ± 2.78	0.50 ± 0.06
	Meliaceae	*Cedrela serrata*	2.84 ± 0.27	0.18 ± 0.00	45.52 ± 0.89	2.05 ± 0.33	0.13 ± 0.01	16.18 ± 2.13	0.38 ± 0.04
	Malvaceae	*Brachychiton acerifolius*	2.48 ± 0.18	0.24 ± 0.08	43.95 ± 0.29	2.28 ± 0.24	0.22 ± 0.05	10.27 ± 2.55	0.45 ± 0.17
Mean			2.15 ± 0.42 ^AB^	0.17 ± 0.07	44.14 ± 0.93 ^A^	2.14 ± 0.17	0.16 ± 0.06	15.09 ± 3.93	0.42 ± 0.04^B^
Asterids	Bignoniaceae	*Jacaranda rnirnosifolia*	2.53 ± 0.25	0.19 ± 0.01	46.40 ± 1.54	3.01 ± 0.78	0.23 ± 0.07	13.07 ± 1.15	0.26 ± 0.03
	Boraginaceae	*Cordia alliodora*	2.81 ± 0.31	0.22 ± 0.04	41.39 ± 0.93	1.75 ± 0.39	0.14 ± 0.03	12.88 ± 3.10	0.16 ± 0.02
	Oleaceae	*Ligustrum lucidum*	2.79 ± 0.45	0.17 ± 0.05	43.14 ± 1.87	2.97 ± 0.34	0.18 ± 0.02	16.30 ± 2.10	0.60 ± 0.04
	Solanaceae	*Cyphornandra betaceae*	4.30 ± 0.34	0.37 ± 0.05	43.03 ± 0.77	2.72 ± 0.28	0.23 ± 0.02	11.65 ± 1.26	0.43 ± 0.07
	Asteraceae	*Tithonia diversifolia*	5.06 ± 0.58	0.34 ± 0.06	41.79 ± 1.54	1.91 ± 0.19	0.13 ± 0.01	15.03 ± 0.21	0.25 ± 0.07
Mean			3.50 ± 0.98^B^	0.26 ± 0.08	43.15 ± 1.73^AB^	2.47 ± 0.53	0.18 ± 0.04	13.79 ± 1.63	0.34 ± 0.15^B^

Group means with different letters for a trait were significantly different (*P* ≤ 0.05)

The values mean ± 95% CI

*N*_*m*_ leaf mass-based N concentration, *P*_*m*_ leaf mass-based P concentration, *C*_*m*_ leaf mass-based C concentration, *N*_*a*_ leaf area-based N content, *P*_*a*_ leaf area-based P content

All these traits varied greatly among individual species and species-specific data can be found in Table 2 and Online Resource 2.

### Functional relationships

**Fig. 4:**
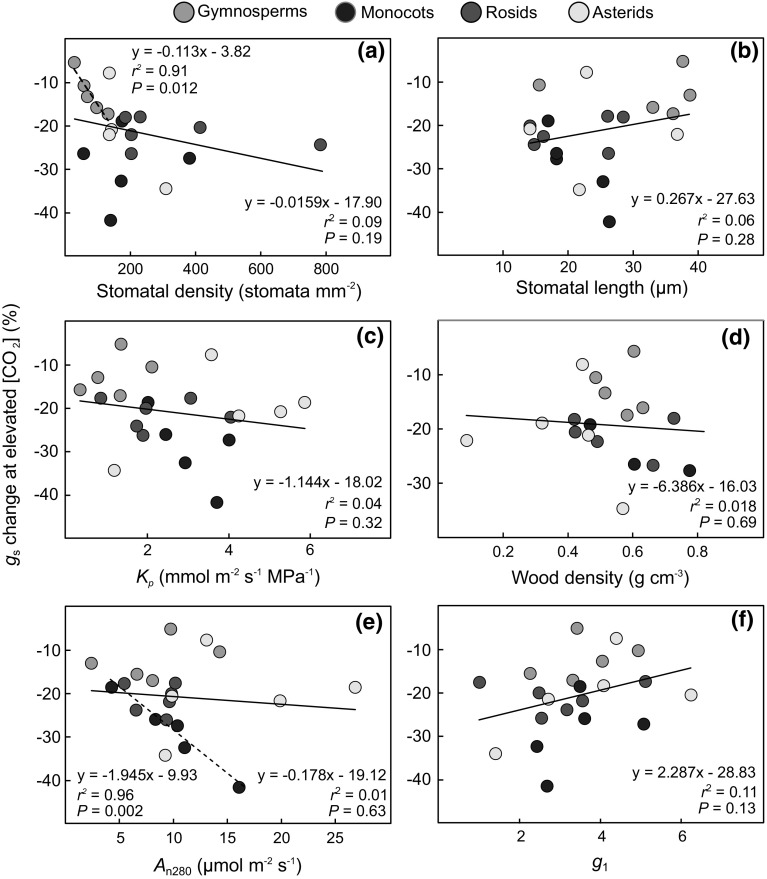
The short-term *g*_s_ response to elevated [CO_2_] (600 vs. 400 µmol mol^−1^) in relation to **a** stomatal density; **b** stomatal length; **c** leaf area-specific plant hydraulic conductance (*K*_p_); **d** wood density; **e** net photosynthetic rate at 280 µmol mol^−1^ intercellular [CO_2_] (*A*_n280_); and **f** *g*_1_ (see Eq. 3). Regression lines with *r* ^2^ and *P* values are shown. *Solid lines* for relationships across all species and *dashed lines* for significant (*P* ≤ 0.05) relationships within individual taxonomic groups (gymnosperms in 4a and monocots in 4e). A color version of this figure is available in the online version of the journal

Across all data, the inter-specific variation in the short-term *g*_s_ response to increased [CO_2_] (i.e. 600 vs. 400 µmol mol^−1^) was not significantly related to variation in stomatal density, stomatal length, *K*_p_, wood density, *A*_n280_ or *g*_1_ (*r* ^2^ ≤ 0.11; *P* ≥ 0.13; Fig. 4a–f). There were, however, a couple of exceptions within individual taxonomic groups, with stomatal CO_2_ responsiveness being stronger in gymnosperm species with high stomatal density (*r* ^2^ = 0.91; *P* = 0.011; Fig. 4a) and in monocot species with high *A*_n280_ (*r* ^2^ = 0.96; *P* = 0.002; Fig. 4e).

**Fig. 5:**
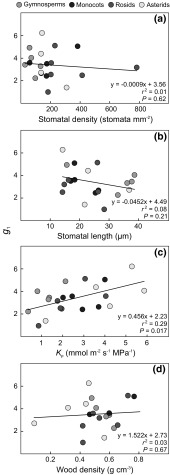
The combined stomatal–photosynthesis model parameter *g*_1_ (see Eq. 3) in relation to **a** stomatal density; **b** stomatal length; **c** leaf area-specific plant hydraulic conductance (*K*_p_); and **d** wood density. Regression lines with *r* ^2^ and *P* values are shown. A color version of this figure is available in the online version of the journal

Values of *g*_1_ showed a positive relationship with *K*_p_ (*r* ^2^ = 0.29; *P* = 0.017), but were not significantly related to stomatal density or length, or wood density (*r* ^2^ < 0.08; *P* > 0.21; Fig. 5).

**Fig. 6:**
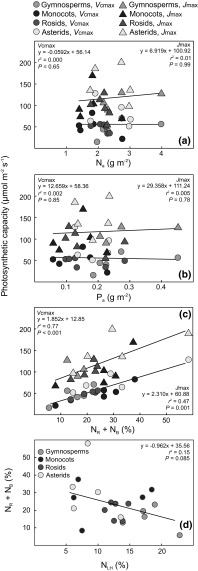
Photosynthetic capacity (i.e. *V*_cmax_ and *J*_max_) in relation to **a** area-based leaf N content (N_a_); **b** area-based leaf P content (P_a_); and **c** the fraction of leaf N allocated to Rubisco and bioenergetics (N_R_ + N_B_). Also shown **d** is the fraction of leaf N allocated to Rubisco and bioenergetics (N_R_ + N_B_) vs. the fraction of N allocated to light harvesting (N_LH_). Regression lines with *r* ^2^ and *P* values are shown. A color version of this figure is available in the online version of the journal

**Fig. 7:**
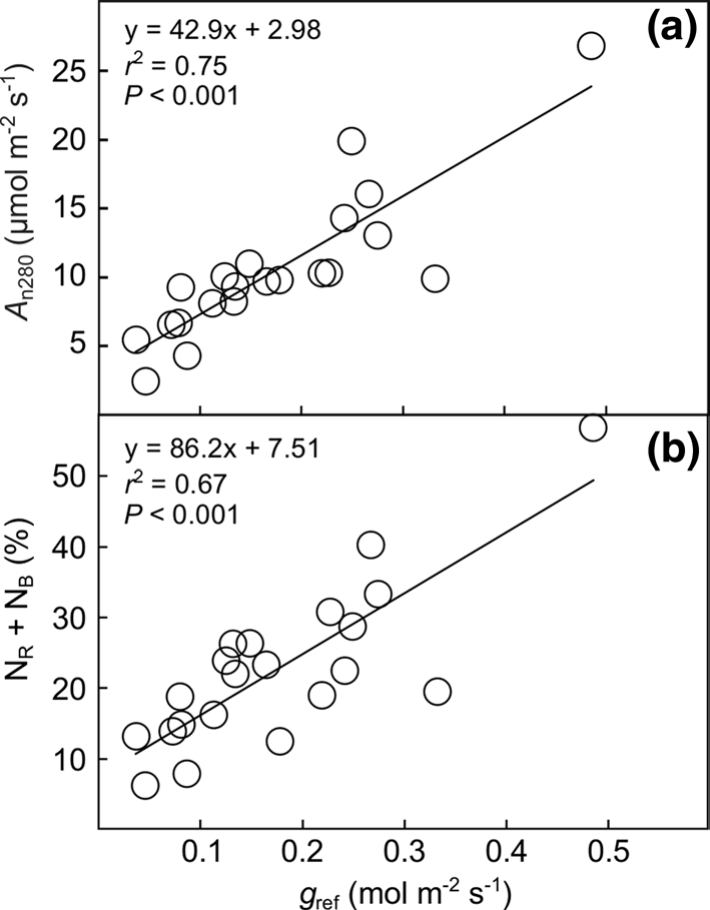
**a** Net photosynthetic rate at 280 µmol mol^−1^ intercellular [CO_2_] (*A*_n280_) and **b** the fraction of leaf N allocated to Rubisco and bioenergetics (N_R_ + N_B_) in relation to stomatal conductance at 400 µmol mol^−1^ air [CO_2_]

Values of *V*_cmax_ and *J*_max_ had no significant relationship with N_a_ (*r* ^2^ < 0.01; *P* ≥ 0.65; Fig. 6a) or P_a_ (*r* ^2^ < 0.004; *P* > 0.78; Fig. 6b), but was significantly related to leaf N_m_ (*r* ^2^ = 0.53; *P* < 0.001 and *r* ^2^ = 0.28; *P* = 0.027, respectively; Online Resource 3a) and P_m_ (*r* ^2^ = 0.40; *P* = 0.002 and *r* ^2^ = 0.29; *P* = 0.015, respectively; Online Resource 3b). The photosynthetic N and P use-efficiencies (i.e. the ratios of *A*_n280_ to N_a_ and P_a_, respectively) did not significantly differ among plant groups, largely due to large interspecific variation (*P* ≥ 0.19; Online Resource 4). The variation in *V*_cmax_ and *J*_max_ among species was, however, significantly related to the fraction of leaf N allocated to Rubisco and bioenergetics (N_R_ + N_B_) (*r* ^2^ = 0.77; *P* < 0.001 and *r* ^2^ = 0.47; *P* = 0.001, respectively; Fig. 6c). In addition, there was a marginally significant negative relationship between N_R_ + N_B_ and the fraction of leaf N allocated to light harvesting (*N*_LH_; *r* ^2^ = 0.15; *P* = 0.085; Fig. 6d). Values of *V*_cmax_ and *J*_max_ were closely related, both within taxonomic groups and across the entire data set (*r* ^2^ = 0.82; *P* < 0.001; Online Resource 5). Finally, both *A*_n280_ (*r* ^2^ = 0.75) and N_R_ + N_B_ (*r* ^2^ = 0.67) were strongly and positively related to *g*_s_ measured at 400 µmol mol^−1^ air [CO_2_] (Fig. 7).

## Discussion

Data on physiological, structural and chemical plant traits were collected and analyzed to explore the controls of interspecific variation in stomatal CO_2_ responsiveness and photosynthetic capacity (*V*_cmax_ and *J*_max_) among tropical woody plant species in central Africa. The broad selection of tree species together with the common garden approach allowed us to isolate the effects of long-term evolutionary adaptation from plastic adjustments to local environmental conditions (i.e., acclimation), a separation that is not possible in observational studies or meta-analyses where different species are studied at different locations and the variation in plant traits is the combined result of both adaptation and acclimation. This study specifically addressed the following three questions:

### Does the short-term stomatal response to [CO_2_] vary among taxonomic groups?

Species of all taxonomic groups significantly decreased *g*_s_ in response to a short-term increase in [CO_2_] (Fig. 2e), with this response being stronger in angiosperms than in gymnosperms and in monocots compared to dicots (Online Resource 1). These results are generally in line with earlier observations of lower stomatal CO_2_ responsiveness in gymnosperm compared to angiosperm species (Medlyn et al. 2001; Brodribb et al. 2009), but do not support the claim that stomata of gymnosperms entirely lack primary responses to increased [CO_2_] (Brodribb et al. 2009). Other recent studies also found significant stomatal closure responses to increased [CO_2_] in gymnosperm species (e.g. Haworth et al. 2013) and molecular studies indicate that the genetic tool kits necessary to respond to environmental cues such as [CO_2_] were present already in early land plants and are not exclusive to angiosperms (Chater et al. 2013). Furthermore, observational studies based on ecosystem flux measurements or stable carbon isotopes in tree rings in temperate and boreal ecosystems have found similar (Keenan et al. 2013) or even stronger (Frank et al. 2015) increases in water-use efficiency under rising atmospheric [CO_2_] in gymnosperms compared to angiosperms, further indicating that species of both taxonomic groups may have stomatal closure responses to increased [CO_2_]. If we assume that short-term stomatal CO_2_ responsiveness is an important determinant of the long-term effect of growth under elevated [CO_2_] on *g*_s_, as indicated by observations in free-air CO_2_ enrichment experiments (Fig. 1), our results suggest that stomatal closure-induced leaf water savings under rising [CO_2_] may be expected in both angiosperms and gymnosperms also in tropical forests.

### What functional characteristics can explain the interspecific variation in stomatal behavior?

Across all species, the interspecific variation in short-term stomatal CO_2_ responsiveness was not significantly related to any of the measured structural or functional plant traits (Fig. 4). However, in gymnosperms, the taxonomic group with the lowest stomatal density, the *g*_s_ response got stronger with increasing stomatal density (Fig. 4a). We also found a significant relationship between *A*_n280_ and stomatal CO_2_ responsiveness in monocots (Fig. 4e), indicating that CO_2_ responsiveness may be linked to the metabolic activity of the leaf in this taxonomic group where stomatal CO_2_ responses were strong (Fig. 2e). Further studies are required to confirm these relationships and why they are not found in all taxonomic groups.

The slope parameter of the combined stomatal-photosynthesis model (Eq. 3), *g*_1_, was significantly related to leaf area-specific plant hydraulic conductance, *K*_p_ (Fig. 5c). Admittedly, the positive relationship found between *g*_1_ and *K*_p_ (Fig. 5c) may be confounded by the use of leaf gas exchange data in the calculation of both traits (see Eqs. 3 and  5). However, we find it highly likely that it reflects a true dependence of the marginal carbon cost of water use, which is inversely related to the square of *g*_1_ according to the model of optimal stomatal behaviour proposed by Medlyn et al. (2011). Such dependence agrees well with the common observation that plants that use a lot of water (which is made possible though high *K*_p_) also have low water-use efficiency (i.e. high *g*_1_; Larcher 2003). We did not, however, find that *g*_1_ was negatively related to wood density (Fig. 5d), as reported for angiosperm trees in a recent global meta-analysis (Lin et al. 2015). The suggestion by Lin et al. (2015) that wood density is a good proxy for quantifying *g*_1_ in angiosperm trees is thus not corroborated by the present study, which points at tree hydraulics being more important. The mean *g*_1_ value for gymnosperms observed in this study (3.45) was not lower than that for angiosperms (3.33; Fig. 2f), and markedly higher than the mean *g*_1_ value for gymnosperms reported by Lin et al. (2015) (2.35). This discrepancy between our study and the Lin et al. meta-analysis may be a consequence of the shortage of tropical gymnosperm data in the latter study, and that gymnosperm and angiosperm trees may have similar *g*_1_ if measured in similar climates (at least in the tropics).

### What controls the interspecific variation in photosynthetic capacity?

Total leaf area-based nutrient content (N_a_ and P_a_) did not explain the large interspecific variation in photosynthetic capacity (Fig. 6a, b), in agreement with previous tropical studies (Coste et al. 2005; van de Weg et al. 2012; Houter and Pons 2014; Dusenge et al. 2015). Instead, the differences in photosynthetic capacities among species were strongly linked to the fractional investment of leaf N into compounds maximizing these capacities, i.e. N_R_ + N_B_ (Fig. 6c). Similar results were reported from two studies with tropical montane tree species (Coste et al. 2005; Dusenge et al. 2015) and in a global meta-analysis (Ali et al. 2015). Dusenge et al. (2015) suggested that there is a trade-off, such that species with large fractional leaf N investment into light harvesting have low investments into compounds maximizing photosynthetic capacity, and vice versa. In that study, which included a limited number of tree species (*n* = 10), the former strategy was present in climax species while the latter strategy was used by pioneers. This study corroborates the hypothesis of Dusenge et al. (2015), with a marginally significant (*P* = 0.08) negative relationship between the relative (%) leaf N investments into Rubisco and bioenergetics (N_R_ + N_B_) versus light harvesting compounds (N_LH_; Fig. 6d). It should be noted that while the relationship between N_R_ + N_B_ and photosynthetic capacity (Fig. 6c) was expected since the absolute N investments into these components were calculated from the values of *V*_cmax_ and *J*_max_ (Eqs. 6 , 7), the relationship between N_LH_ and N_R_ + N_B_ (Fig. 6d) was based on independent estimates of leaf chlorophyll content and photosynthetic capacity, respectively.

Leaf N allocation and water use were strongly co-ordinated (*r* ^2^ = 0.67; Fig. 7b), showing that species with high relative N investments into compounds maximizing photosynthetic capacity take advantage of this investment by having high *g*_s_. This finding is in line with earlier observations of tight co-ordination between photosynthesis and *g*_s_ (e.g. Wong et al. 1979; Franks and Farquhar 1999), but extends beyond these by showing that also within-leaf N allocation strategy plays an important part in this coupling.

These results demonstrate that the interspecific variation in photosynthetic capacity is strongly linked to within-leaf N allocation and water use strategies in tropical woody species. The finding that within-leaf N allocation is more important than total leaf N content in controlling photosynthetic capacity implies that current vegetation models, which often assume that *V*_cmax_ and *J*_max_ are plant functional type-specific functions of area-based leaf N content (e.g. Rogers 2014; Zaehle et al. 2005), would be much improved if they could also account for differences in within-leaf N allocation.

## Electronic supplementary material

Below is the link to the electronic supplementary material.

Supplementary material 1 (PDF 402 kb)

Supplementary material 2 (PDF 800 kb)

Supplementary material 3 (PDF 392 kb)

Supplementary material 4 (PDF 678 kb)

Supplementary material 5 (PDF 387 kb)
