# Supplementary material for: Stomatal CO2 responsiveness and photosynthetic capacity of tropical woody species in relation to taxonomy and functional traits
Source: Oecologia. 2017 Mar 4;184(1):43–57. doi: 10.1007/s00442-017-3829-0 (PMC5408058; doi:10.1007/s00442-017-3829-0)
Supplement: Supplementary file 1 — Supplementary material 1 (PDF 402 kb) [file 442_2017_3829_MOESM1_ESM.pdf]

Oecologia

Electronic Supplemental Material (ESM)

Online Resource 1. Comparison of short-term stomatal responses to increased CO_2 between (a) Gymnosperms and Angiosperms and (b) Monocots and Dicots. Error bars represent standard errors of the means. *P* values are shown.

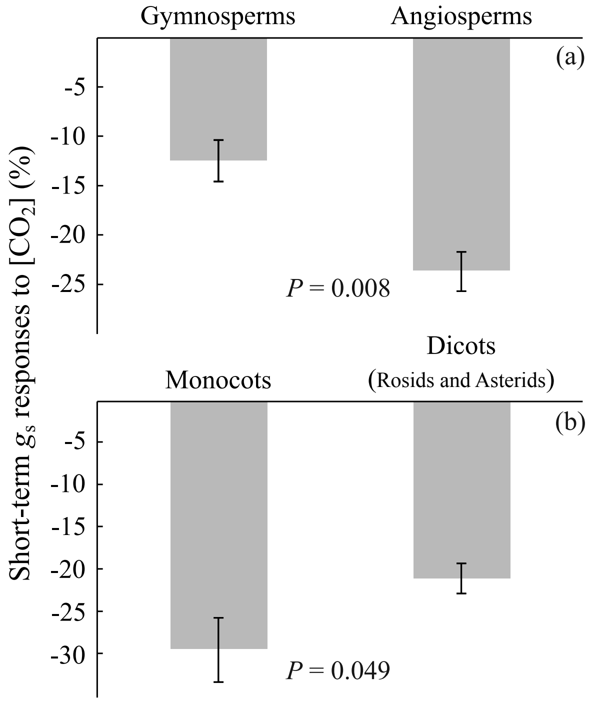

Stomatal CO_2 responsiveness and photosynthetic capacity of tropical woody species in relation to taxonomy and functional traits

Thomas B. Hasper, Mirindi E. Dusenge, Friederike Breuer, Felicien U. Félicien K. Uwizeye, Göran Wallin, Johan Uddling
