# Supplementary material for: Stomatal CO2 responsiveness and photosynthetic capacity of tropical woody species in relation to taxonomy and functional traits
Source: Oecologia. 2017 Mar 4;184(1):43–57. doi: 10.1007/s00442-017-3829-0 (PMC5408058; doi:10.1007/s00442-017-3829-0)
Supplement: Supplementary file 2 — Supplementary material 2 (PDF 800 kb) [file 442_2017_3829_MOESM2_ESM.pdf]

Oecologia
Electronic Supplemental Material (ESM)

Online Resource 2. Leaf traits among species and groups (means ± CI, 95%; g_s = stomatal conductance; A_{n280} = light-saturated net photosynthesis at an intercellular [CO_2] of 280 µmol mol^{-1}; V_{cmax} = maximum rates of photosynthetic carboxylation; J_{max} = maximum rates of electron transport; g_1 = parameter describing the relationship between photosynthesis and stomatal conductance in combined leaf gas exchange models (see Eq 3); WUE = water use efficiency; K_p = leaf area-specific plant hydraulic conductance; LMA = leaf mass area; g_{smax} = maximum stomatal conductance calculated from stomatal density and dimensions; N_R = fractional leaf N invested in rubisco; N_B = fractional leaf N invested in bioenergetics; N_{LH} = fractional leaf N invested in light harvesting).

Group	Family	Species	**g_s at ambient [CO_2] (mol m^{-2} s^{-1})**	**g_s change at elevated [CO_2] (%)**	**A_{n280} (µmol m^{-2} s^{-1})**	**V_{cmax} (µmol m^{-2} s^{-1})**	**J_{max} (µmol m^{-2} s^{-1})**	**g_1**	**WUE (mmol CO_2 mol H2O^{-1})**
**Gymnosperms**	Araucariaceae	*Araucaria angustifolia*	0.047 ± 0.020	-13.1 ± 10.2	2.4 ± 1.2	16.1 ± 4.4	n ± n	4.04 ± 1.61	3.92 ± 1.29
	Cupressacaea	*Cupressus lusitanica*	0.181 ± 0.061	-5.4 ± 7.7	9.8 ± 1.7	56.4 ± 6.9	129.2 ± 19.2	3.44 ± 0.99	4.46 ± 0.88
	Pinaceae	*Pinus patula*	0.245 ± 0.077	-10.6 ± 7.1	14.3 ± 3.0	70.5 ± 12.5	131.6 ± 24.4	4.93 ± 1.77	4.38 ± 0.56
	Podocarpaceae	*Podocarpus latifolius*	0.112 ± 0.032	-17.3 ± 12.1	8.1 ± 2.2	41.0 ± 10.0	92.1 ± 12.0	3.32 ± 0.49	3.91 ± 0.28
	Podocarpaceae	*Podocarpus falcatus*	0.081 ± 0.022	-15.8 ± 16.0	6.6 ± 1.1	44.7 ± 10.7	108.2 ± 21.9	2.25 ± 0.71	5.29 ± 0.79
		Mean	**0.133 ± 0.070**	**-12.4 ± 4.1**	**8.2 ± 3.8**	**45.7 ± 17.7**	**115.3 ± 18.3**	**3.59 ± 0.86**	**4.39 ± 0.49**
**Monocots**	Arecaceae	*Phoenix reclinata*	0.089 ± 0.044	-18.8 ± 17.9	4.2 ± 2.3	22.1 ± 10.3	71.2 ± 23.3	3.49 ± 2.22	4.15 ± 1.28
	Poaceae	*Dendrocalamus giganteus*	0.268 ± 0.083	-27.4 ± 6.2	10.4 ± 3.5	52.9 ± 13.5	93.2 ± 28.2	5.07 ± 1.83	3.09 ± 0.26
	Poaceae	*Bambusa vulgaris*	0.134 ± 0.022	-26.2 ± 8.8	8.3 ± 0.6	42.7 ± 5.4	76.5 ± 10.0	3.60 ± 0.68	4.09 ± 0.96
	Heliconiaceae	*Heliconia sp.*	0.215 ± 0.119	-41.7 ± 8.0	11.0 ± 3.2	57.1 ± 10.4	106.1 ± 15.4	2.43 ± 0.63	4.92 ± 1.08
	Musacea	*Musa sapientum*	0.235 ± 0.093	-32.7 ± 17.9	16.0 ± 5.6	82.2 ± 10.5	171.9 ± 21.5	2.67 ± 1.31	7.88 ± 3.95
		Mean	**0.188 ± 0.065**	**-29.4 ± 7.5**	**10.0 ± 3.8**	**51.4 ± 19.2**	**103.8 ± 35.5**	**3.45 ± 0.91**	**4.83 ± 1.60**
**Rosids**	Chrysobalanaceae	*Macaranga kilimandscharica*	0.167 ± 0.043	-22.2 ± 3.0	9.6 ± 3.4	52.5 ± 13.6	103.8 ± 19.7	3.56 ± 0.53	3.94 ± 0.47
	Rosaceae	*Prunus careta*	0.220 ± 0.043	-17.8 ± 14.7	10.3 ± 2.2	49.3 ± 10.0	95.6 ± 11.2	5.11 ± 1.12	3.46 ± 0.69
	Myrtaceae	*Eucalyptus maculata*	0.138 ± 0.053	-26.3 ± 7.0	9.4 ± 2.0	54.1 ± 8.2	131.7 ± 26.8	2.54 ± 0.70	5.25 ± 0.56
	Meliaceae	*Carapa grandiflora*	0.073 ± 0.017	-24.2 ± 13.8	6.5 ± 3.0	34.6 ± 13.7	84.9 ± 16.1	3.17 ± 1.85	4.97 ± 1.51
	Meliaceae	*Cedrela serrate*	0.126 ± 0.019	-20.2 ± 10.3	9.9 ± 1.8	56.3 ± 11.4	115.8 ± 17.3	2.49 ± 0.31	5.98 ± 1.53
	Malvaceae	*Brachychiton acerifolius*	0.040 ± 0.011	-17.8 ± 6.2	5.5 ± 1.6	35.3 ± 9.0	56.8 ± 17.3	1.00 ± 0.42	7.66 ± 1.41
		Mean	**0.127 ± 0.052**	**-21.4 ± 2.7**	**8.5 ± 1.6**	**47.0 ± 7.7**	**98.1 ± 20.7**	**2.98 ± 1.09**	**5.21 ± 1.20**
**Asterids**	Bignoniaceae	*Jacaranda mimosifolia*	0.318 ± 0.074	-20.8 ± 10.8	9.9 ± 3.3	68.1 ± 17.4	138.3 ± 40.7	6.24 ± 1.77	2.56 ± 0.47
	Boraginaceae	*Cordia alliodora*	0.344 ± 0.265	-7.8 ± 27.8	13.1 ± 4.4	64.1 ± 21.8	137.4 ± 53.7	4.39 ± 0.86	3.80 ± 0.62
	Oleaceae	*Ligustrum lucidum*	0.087 ± 0.041	-34.4 ± 7.1	9.2 ± 3.5	51.6 ± 20.2	99.9 ± 22.7	1.40 ± 0.67	7.39 ± 1.04
	Solanaceae	*Cyphomandra betaceae*	0.242 ± 0.056	-21.8 ± 16.6	19.9 ± 2.8	96.1 ± 13.0	201.9 ± 10.9	2.70 ± 1.03	4.79 ± 0.50
	Asteraceae	*Tithonia diversifolia*	0.476 ± 0.073	-18.7 ± 11.9	26.8 ± 0.9	128.6 ± 4.9	188.1 ± 14.2	4.08 ± 0.70	4.71 ± 0.46
		Mean	**0.293 ± 0.125**	**-20.7 ± 8.3**	**15.8 ± 6.6**	**81.7 ± 27.0**	**153.1 ± 36.4**	**3.76 ± 1.60**	**4.65 ± 1.56**

n = Not measured

Stomatal CO_2 responsiveness and photosynthetic capacity of tropical woody species in relation to taxonomy and functional traits
Thomas B. Hasper, Mirindi E. Dusenge, Friederike Breuer, Felicien U. Félicien K. Uwizeye, Göran Wallin, Johan Uddling

Oecologia
Electronic Supplemental Material (ESM)

Continuation

Group	Family	Species	K_p (mmol m^{-2} s^{-1} MPa^{-1})	Stomata density (stomata mm^{-2})	Stomatal length (µm)	Wood density (g cm^{-3})	LMA (g m^{-2})	g_{smax} (mol m^{-2} s^{-1})	N_R (%)	N_B (%)	N_{LH} (%)
**Gymnosperms**	Araucariaceae	*Araucaria angustifolia*	0.78 ± 0.37	68.4 ± 3.2	38.63 ± 2.76	0.51 ± 0.03	119.0 ± 18.7	1.12 ± 0.09	5.5 ± 3.6	n ± n	22.9 ± 4.8
	Cupressacaea	*Cupressus lusitanica*	1.35 ± 0.68	27.0 ± 4.2	37.58 ± 5.26	0.60 ± 0.07	315.9 ± 23.6	0.43 ± 0.12	10.3 ± 1.5	2.62 ± 0.49	13.9 ± 2.4
	Pinaceae	*Pinus patula*	2.12 ± 0.91	57.0 ± 2.4	15.64 ± 3.14	0.48 ± 0.05	172.5 ± 10.0	0.38 ± 0.04	19.3 ± 2.7	3.79 ± 0.30	19.1 ± 8.3
	Podocarpaceae	*Podocarpus latifolius*	1.32 ± 0.13	131.3 ± 8.2	36.16 ± 3.61	0.58 ± 0.04	141.8 ± 28.2	2.01 ± 0.29	13.1 ± 1.3	3.51 ± 0.68	17.4 ± 4.9
	Podocarpaceae	*Podocarpus falcatus*	0.34 ± 0.09	96.4 ± 5.7	32.98 ± 3.63	0.63 ± 0.25	120.7 ± 11.2	1.35 ± 0.18	15.4 ± 3.3	4.03 ± 0.76	17.1 ± 3.9
		Mean	**1.18 ± 0.59**	**76.0 ± 34.8**	**32.20 ± 8.33**	**0.56 ± 0.05**	**174.0 ± 72.1**	**1.06 ± 0.60**	**12.7 ± 4.6**	**3.49 ± 0.61**	**18.1 ± 2.9**
**Monocots**	Arecaceae	*Phoenix reclinata*	2.02 ± 1.32	174.9 ± 7.0	16.92 ± 1.83	0.47 ± 0.03	144.6 ± 7.6	1.25 ± 0.11	6.0 ± 2.6	2.05 ± 0.60	7.5 ± 3.5
	Poaceae	*Dendrocalamus giganteus*	4.01 ± 1.85	381.0 ± 7.6	18.19 ± 1.83	0.77 ± 0.12	39.6 ± 9.3	2.94 ± 1.18	26.3 ± 1.7	5.24 ± 0.45	18.5 ± 3.0
	Poaceae	*Bambusa vulgaris*	2.44 ± 0.56	57.1 ± 3.5	18.20 ± 2.83	0.60 ± 0.17	47.9 ± 7.5	0.44 ± 0.06	23.0 ± 1.7	4.30 ± 0.46	17.1 ± 2.8
	Heliconiaceae	*Heliconia sp.*	2.93 ± 0.95	171.6 ± 5.0	25.40 ± 2.18	n ± n	60.7 ± 5.0	1.85 ± 0.05	22.4 ± 2.9	4.57 ± 0.54	6.2 ± 0.7
	Musacea	*Musa sapientum*	3.70 ± 1.50	138.1 ± 3.4	26.33 ± 3.38	n ± n	52.9 ± 10.4	1.54 ± 0.13	30.7 ± 6.0	6.96 ± 0.68	7.0 ± 1.3
		Mean	**3.02 ± 0.73**	**184.5 ± 104.9**	**21.01 ± 3.92**	**0.61 ± 0.17**	**69.1 ± 37.6**	**1.60 ± 0.80**	**21.7 ± 8.2**	**4.63 ± 1.55**	**11.2 ± 5.3**
**Rosids**	Chrysobalanaceae	*Macaranga kilimandscharica*	4.04 ± 2.32	203.1 ± 3.3	16.19 ± 1.81	0.49 ± 0.04	85.3 ± 12.7	1.39 ± 0.50	18.9 ± 3.6	4.67 ± 0.60	14.8 ± 2.9
	Rosaceae	*Prunus careta*	3.05 ± 0.92	228.7 ± 10.2	26.10 ± 2.24	0.72 ± 0.05	98.9 ± 23.3	2.53 ± 0.32	16.5 ± 1.6	3.59 ± 0.31	11.1 ± 3.2
	Myrtaceae	*Eucalyptus maculata*	1.88 ± 0.81	202.1 ± 4.1	26.27 ± 3.50	0.66 ± 0.02	170.6 ± 22.7	2.25 ± 0.24	19.0 ± 3.9	4.99 ± 0.46	12.8 ± 2.7
	Meliaceae	*Carapa grandiflora*	1.74 ± 1.07	783.3 ± 5.2	14.78 ± 2.25	0.53 ± 0.05	118.3 ± 13.8	4.91 ± 0.66	11.1 ± 3.6	2.69 ± 0.40	14.0 ± 2.0
	Meliaceae	*Cedrela serrate*	1.94 ± 0.63	413.5 ± 6.6	14.18 ± 1.71	0.42 ± 0.03	72.1 ± 7.8	2.48 ± 0.89	19.8 ± 1.3	4.49 ± 0.11	12.2 ± 1.5
	Malvaceae	*Brachychiton acerifolius*	0.86 ± 0.24	184.2 ± 10.8	28.43 ± 2.62	0.42 ± 0.03	92.0 ± 9.9	2.22 ± 0.35	11.7 ± 3.1	2.04 ± 0.56	12.5 ± 4.3
		Mean	**2.25 ± 0.90**	**335.8 ± 188.1**	**20.99 ± 5.28**	**0.54 ± 0.10**	**106.2 ± 28.0**	**2.63 ± 0.95**	**16.2 ± 3.1**	**3.74 ± 0.95**	**12.9 ± 1.0**
**Asterids**	Bignoniaceae	*Jacaranda mimosifolia*	5.28 ± 0.90	142.5 ± 3.0	14.14 ± 2.89	0.46 ± 0.04	119.2 ± 26.1	0.85 ± 0.07	17.2 ± 2.6	3.76 ± 0.43	6.3 ± 2.9
	Boraginaceae	*Cordia alliodora*	3.57 ± 0.90	135.0 ± 8.6	22.86 ± 4.03	0.44 ± 0.04	62.1 ± 7.3	1.31 ± 0.55	27.4 ± 7.4	6.05 ± 1.66	6.1 ± 0.8
	Oleaceae	*Ligustrum lucidum*	1.18 ± 0.69	308.8 ± 4.9	21.70 ± 2.03	0.56 ± 0.02	106.4 ± 21.3	2.84 ± 0.22	13.0 ± 4.9	2.76 ± 0.63	13.6 ± 2.5
	Solanaceae	*Cyphomandra betaceae*	4.24 ± 1.13	136.9 ± 5.9	36.79 ± 3.71	0.09 ± 0.01	63.3 ± 5.0	2.13 ± 0.54	24.4 ± 4.3	5.96 ± 0.60	10.0 ± 1.0
	Asteraceae	*Tithonia diversifolia*	5.87 ± 1.08	n ± n	n ± n	0.32 ± 0.08	37.6 ± 4.8	n ± n	51.7 ± 7.0	7.96 ± 0.50	8.5 ± 2.2
		Mean	**4.03 ± 1.60**	**180.8 ± 83.7**	**23.87 ± 9.25**	**0.37 ± 0.16**	**77.7 ± 29.7**	**1.78 ± 0.86**	**26.7 ± 13.2**	**5.30 ± 1.80**	**8.9 ± 2.7**

n = Not measured

Stomatal CO_2 responsiveness and photosynthetic capacity of tropical woody species in relation to taxonomy and functional traits
Thomas B. Hasper, Mirindi E. Dusenge, Friederike Breuer, Felicien U. Félicien K. Uwizeye, Göran Wallin, Johan Uddling
