# Supplementary material for: Stomatal CO2 responsiveness and photosynthetic capacity of tropical woody species in relation to taxonomy and functional traits
Source: Oecologia. 2017 Mar 4;184(1):43–57. doi: 10.1007/s00442-017-3829-0 (PMC5408058; doi:10.1007/s00442-017-3829-0)
Supplement: Supplementary file 3 — Supplementary material 3 (PDF 392 kb) [file 442_2017_3829_MOESM3_ESM.pdf]

Oecologia

Electronic Supplemental Material (ESM)

Online Resource 3. Photosynthetic capacity (i.e. V_{cmax} and J_{max}) in relation to (a) mass-based leaf N concentration (N_m) and (b) mass-based leaf P concentration (P_m). Regression lines with r^2 and P values are shown.

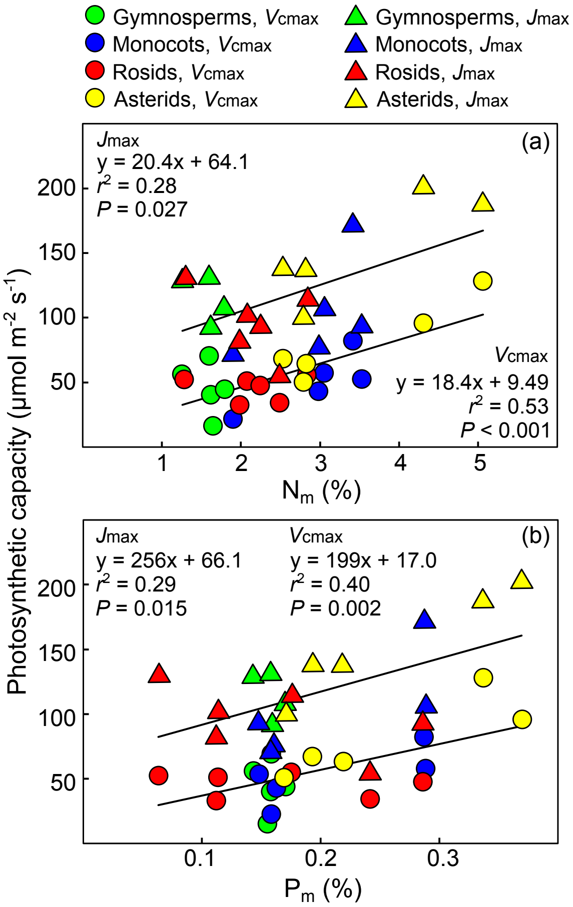

Stomatal CO_2 responsiveness and photosynthetic capacity of tropical woody species in relation to taxonomy and functional traits

Thomas B. Hasper, Mirindi E. Dusenge, Friederike Breuer, Felicien U. Félicien K. Uwizeye, Göran Wallin, Johan Uddling
