# Supplementary material for: Stomatal CO2 responsiveness and photosynthetic capacity of tropical woody species in relation to taxonomy and functional traits
Source: Oecologia. 2017 Mar 4;184(1):43–57. doi: 10.1007/s00442-017-3829-0 (PMC5408058; doi:10.1007/s00442-017-3829-0)
Supplement: Supplementary file 4 — Supplementary material 4 (PDF 678 kb) [file 442_2017_3829_MOESM4_ESM.pdf]

Oecologia

Electronic Supplemental Material (ESM)

Online Resource 4. Photosynthetic N (a) and P (b) use efficiency of different taxonomic groups, expressed as the ratio of light-saturated net photosynthetic rate at 280 µmol mol^{-1} intercellular [CO_2] (A_{n280}) to area-based leaf N and P content, respectively. Each data point represents the mean value of a species and thick black lines represent mean values of the taxonomic groups. GYM, gymnosperms; MON, monocots; ROS, rosids; and AST, asterids.

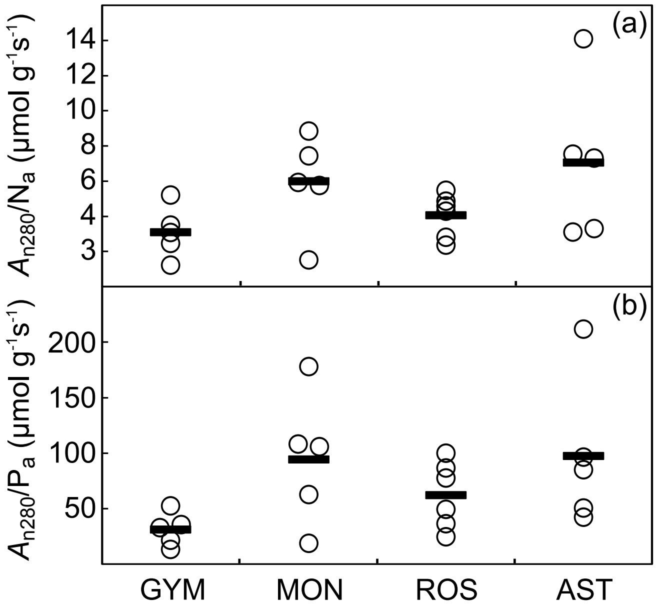

Stomatal CO_2 responsiveness and photosynthetic capacity of tropical woody species in relation to taxonomy and functional traits

Thomas B. Hasper, Mirindi E. Dusenge, Friederike Breuer, Felicien U. Félicien K. Uwizeye, Göran Wallin, Johan Uddling
