# Supplementary material for: Stomatal CO2 responsiveness and photosynthetic capacity of tropical woody species in relation to taxonomy and functional traits
Source: Oecologia. 2017 Mar 4;184(1):43–57. doi: 10.1007/s00442-017-3829-0 (PMC5408058; doi:10.1007/s00442-017-3829-0)
Supplement: Supplementary file 5 — Supplementary material 5 (PDF 387 kb) [file 442_2017_3829_MOESM5_ESM.pdf]

Oecologia

Electronic Supplemental Material (ESM)

Online Resource 5. The relationship between the maximum rates of photosynthetic carboxylation (V_{cmax}) and electron transport (J_{max}) at 25 ºC. Regression line with r^2 and P values is shown.

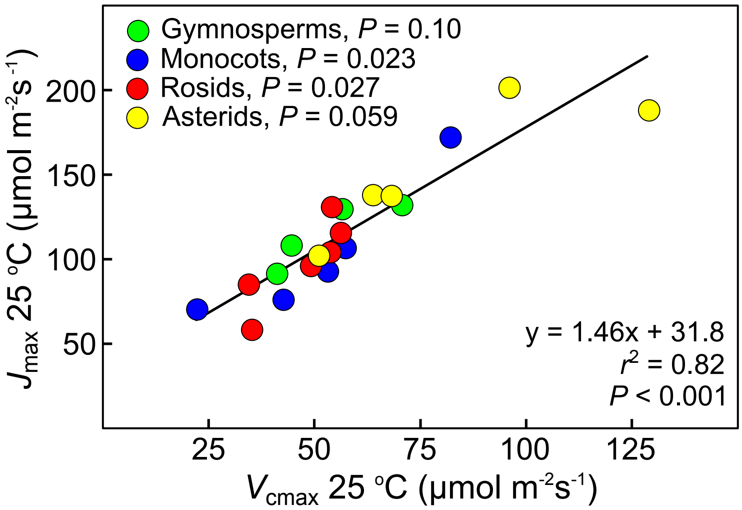

Stomatal CO_2 responsiveness and photosynthetic capacity of tropical woody species in relation to taxonomy and functional traits

Thomas B. Hasper, Mirindi E. Dusenge, Friederike Breuer, Felicien U. Félicien K. Uwizeye, Göran Wallin, Johan Uddling
